# Mixed adenoneuroendocrine carcinoma of the gallbladder: a case report and literature review

**DOI:** 10.3389/fonc.2026.1872890

**Published:** 2026-06-29

**Authors:** Zhenghui Sui, Wei Wang, De Zhang, Yifan Sun, Jiajing Li, Xingwei Gu, Ganggang Miao

**Affiliations:** 1 Department of General Surgery, The People’s Hospital of Danyang, Affiliated Danyang Hospital of Nantong University, Danyang, Jiangsu, China; 2 Department of Basic Medical Sciences, Shanxi Medical University, Taiyuan, Shanxi, China; 3 Department of Surgery, Universitätsklinikum Erlangen, Friedrich-Alexander Universität Erlangen-Nürnberg, Erlangen, Germany; 4 School of Medicine, Jiangsu University, Zhenjiang, Jiangsu, China; 5 School of Clinical Medicine, Wannan Medical College, Wuhu, Anhui, China; 6 Department of General Surgery, The Hospital of Nanjing Qixia District, Nanjing, Jiangsu, China

**Keywords:** gallbladder adenocarcinoma (GB-AC), gallbladder mixed adenoneuroendocrine carcinoma (GB-MANEC), gallbladder neuroendocrine neoplasms (GB-NENs), neuroendocrine neoplasms (NENs), radical surgery

## Abstract

**Background:**

Primary neuroendocrine neoplasms (NENs) of the gallbladder are rare entities with a generally unfavorable prognosis. Among the subtypes of gallbladder neuroendocrine neoplasms (GB-NENs), gallbladder mixed adenoneuroendocrine carcinoma (GB-MANEC) is exceptionally rare and associated with a poor prognosis, representing a diagnostic and therapeutic challenge due to its dual differentiation. This article presents a rare case of GB-MANEC and reviews the pertinent literature on clinicopathological management in GB-MANEC.

**Case presentation:**

A 51-year-old female patient presented with non-specific abdominal complaints and unexplained anemia. Preoperative imaging with abdominal ultrasonography and contrast-enhanced computed tomography (CT) demonstrated an irregular mass within the gallbladder lumen accompanied by wall thickening. The level of carbohydrate antigen 125 (CA125) was mildly elevated (77.10 U/mL; normal level, 0.0–35.0 U/mL). A gallbladder mass measuring approximately 3.0 cm × 2.5 cm × 2.0 cm was identified during the cholecystectomy. Intraoperative frozen section revealed findings consistent with gallbladder adenocarcinoma (GB-AC) accompanied by a component of neuroendocrine carcinoma (NEC). Consequently, a radical surgery (cholecystectomy, partial liver resection, and regional lymphadenectomy) was undertaken. Postoperative histopathological examination confirmed the diagnosis of a GB-MANEC, specifically a tubular adenocarcinoma combined with a NEC, and each component accounted for more than 30%, which was confirmed by immunohistochemistry, represented as positive for synaptophysin (Syn), chromogranin A (CgA), and Ki-67 (90%). Critically, all surgical margins were free of tumor involvement. Postoperative high-throughput sequencing of the tumor identified a pathogenic amplification of the gene ERBB2 Human Epidermal Growth Factor Receptor 2 (HER2); gene mutations of TP53, NOTCH1, and KIT; and a gene fusion of SDHC. Based on these findings, adjuvant chemotherapy was initiated with a combination of cisplatin, gemcitabine, and etoposide, alongside the HER2-targeted monoclonal antibody trastuzumab. During 7 months of postoperative follow-up, imaging studies and blood biochemical assessments showed no evidence of recurrence or metastasis, and the patient has remained free of recurrence.

**Conclusion:**

This systematic review underscores the rarity and aggressive nature of GB-MANEC. A definitive diagnosis is primarily established through postoperative histopathology and immunohistochemistry. Surgical treatment remains the cornerstone of potentially curative treatment, while the dismal prognosis is often due to the high aggressiveness. Currently, the role of adjuvant chemotherapy remains undefined due to the extreme rarity of MANEC, thus highlighting the critical need for a multidisciplinary approach and further research to establish standardized postoperative management guidelines for the diagnosis and management of GB-MANEC.

## 1 Introduction

Gallbladder cancer (GBC) is the most common malignancy of the biliary tract, characterized by its aggressive biological behavior and poor prognosis (1). The vast majority of GBCs are biliary-type adenocarcinomas (1, 2). However, a rare and distinct subset of GBCs, gallbladder neuroendocrine neoplasms (GB-NENs), accounts for 0.5% of all NENs and 2.1% of all gallbladder cancers (3, 4).

NENs are classified into well-differentiated neuroendocrine tumors (NETs), poorly differentiated neuroendocrine carcinomas (NECs), and mixed neuroendocrine non-neuroendocrine neoplasms (MiNENs) (5). Among the subtypes of MiNENs, gallbladder mixed adenoneuroendocrine carcinoma (GB-MANEC) is particularly rare and is characterized by the coexistence of recognizable adenocarcinoma and NEC components, with each component accounting for at least 30% of the tumor, as defined by the World Health Organization (WHO) classification system (6, 7).

Unlike conventional gallbladder adenocarcinoma, GB-MANEC exhibits aggressive biological behavior with a high propensity for early metastasis (8, 9), yet its preoperative diagnosis remains challenging due to non-specific clinical and imaging findings. The coexistence of two distinct malignant components complicates histopathological interpretation and frequently leads to misdiagnosis (10–12). Furthermore, no standardized treatment guidelines exist for GB-MANEC, with current management strategies largely extrapolated from other biliary neuroendocrine or adenocarcinoma tumors (11, 13, 14).

Due to the diagnostic and therapeutic difficulties, documenting individual cases is crucial to expand clinicopathological knowledge and explore optimal management. Herein, we report a case of primary GB-MANEC treated with radical surgical resection followed by adjuvant chemotherapy combined with targeted therapy. Encouragingly, the patient has remained free of recurrence during 7 months of postoperative follow-up, supported by imaging studies and blood biochemical assessments showing no evidence of recurrence or metastasis. This case provides valuable insights into the potential benefit of radical surgery and multimodal adjuvant therapy in achieving short-term disease control for this rare aggressive tumor and highlights the importance of long-term surveillance to define durable outcomes.

## 2 Case presentation

### 2.1 Patient information and clinical presentation

A 51-year-old woman with a Body Mass Index (BMI) of 24.56 presented to our department with non-specific abdominal complaints accompanied by unexplained anemia. The primary symptoms and physical examination revealed no significant findings, and Murphy’s sign was negative.

### 2.2 Diagnostic investigations

#### 2.2.1 Laboratory tests

Laboratory investigations revealed moderate anemia, as evidenced by a low hemoglobin level (65 g/L, normal level, 115–150 g/L), which showed mild improvement following the surgical procedure. Other hematological and biochemical parameters were within normal limits. Regarding the tumor biomarkers, serum carbohydrate antigen 125 (CA125) was mildly elevated (77.10 U/mL, normal level, 0.0–35.0 U/mL) and subsequently normalized following the surgical procedure. Other tumor markers, including carbohydrate antigen 19-9 (CA19-9), carcinoembryonic antigen (CEA), alpha-fetoprotein (AFP), and squamous cell carcinoma-related antigen (SCC-Ag), were normal (**Table 1**).

**Table 1:** The basic information and hematological/biochemical results on admission and during postoperative follow-up (three cycles after adjuvant chemotherapy).

Parameters	On admission	Postoperative follow-up	Reference interval
BMI	24.56	23.72	--
White cell count	4.41 × 10^9^	0.87 × 10^9^	3.5–9.5 × 10^9^/L
Neutrophils	2.60 × 10^9^	0.14 × 10^9^	1.8–6.3 × 10^9^/L
Lymphocytes	1.46 × 10^9^	0.67 × 10^9^	1.1–3.2 × 10^9^/L
Monocytes	0.28 × 10^9^	0.02 × 10^9^	0.1–0.6 × 10^9^/L
Hemoglobin	65 g/L↓	83 g/L↓	115–150 g/L
Platelet	566 × 10^9^↑	31 × 10^9^↓	125–350 × 10^9^/L
Potassium	4.15 mmol/L	4.08 mmol/L	3.5–5.3 mmol/L
Sodium	140.4 mmol/L	142.9 mmol/L	137–147 mmol/L
Chlorine	106.8 mmol/L	106.8 mmol/L	99–110 mmol/L
Urea nitrogen	4.34 mmol/L	6.25 mmol/L	2.6–7.5 mmol/L
Creatinine	49.18 μmol/L	45.6 μmol/L	41–73 μmol/L
Total bilirubin	7.6 μmol/L	4.9 μmol/L	0–21 μmol/L
Direct bilirubin	2.5 μmol/L	1.2 μmol/L	0–7 μmol/L
Alkaline phosphatase	75.7 U/L	135.3 U/L↑	50–135 U/L
Gamma-glutamyl transferase	15.0 U/L	55.1 U/L↑	7–45 U/L
Alanine transaminase	11.1 U/L	29.3 U/L	7–40 U/L
Aspartate transaminase	15.5 U/L	29.0 U/L	13–35 U/L
Cancer antigen 125	77.10 U/mL↑	13.76 U/mL	0–35 U/mL
Cancer antigen 19-9	20.23 U/mL	18.07 U/mL	0–27 U/mL
Carcinoembryonic antigen	1.28 ng/mL	0.99 ng/mL	0–5 ng/mL
Alpha-fetoprotein	2.32 ng/mL	--	0–7 ng/mL
Squamous cell carcinoma-related antigen	0.697 ng/mL	--	0.5–3 ng/mL

#### 2.2.2 Imaging studies

**Figure 1:**
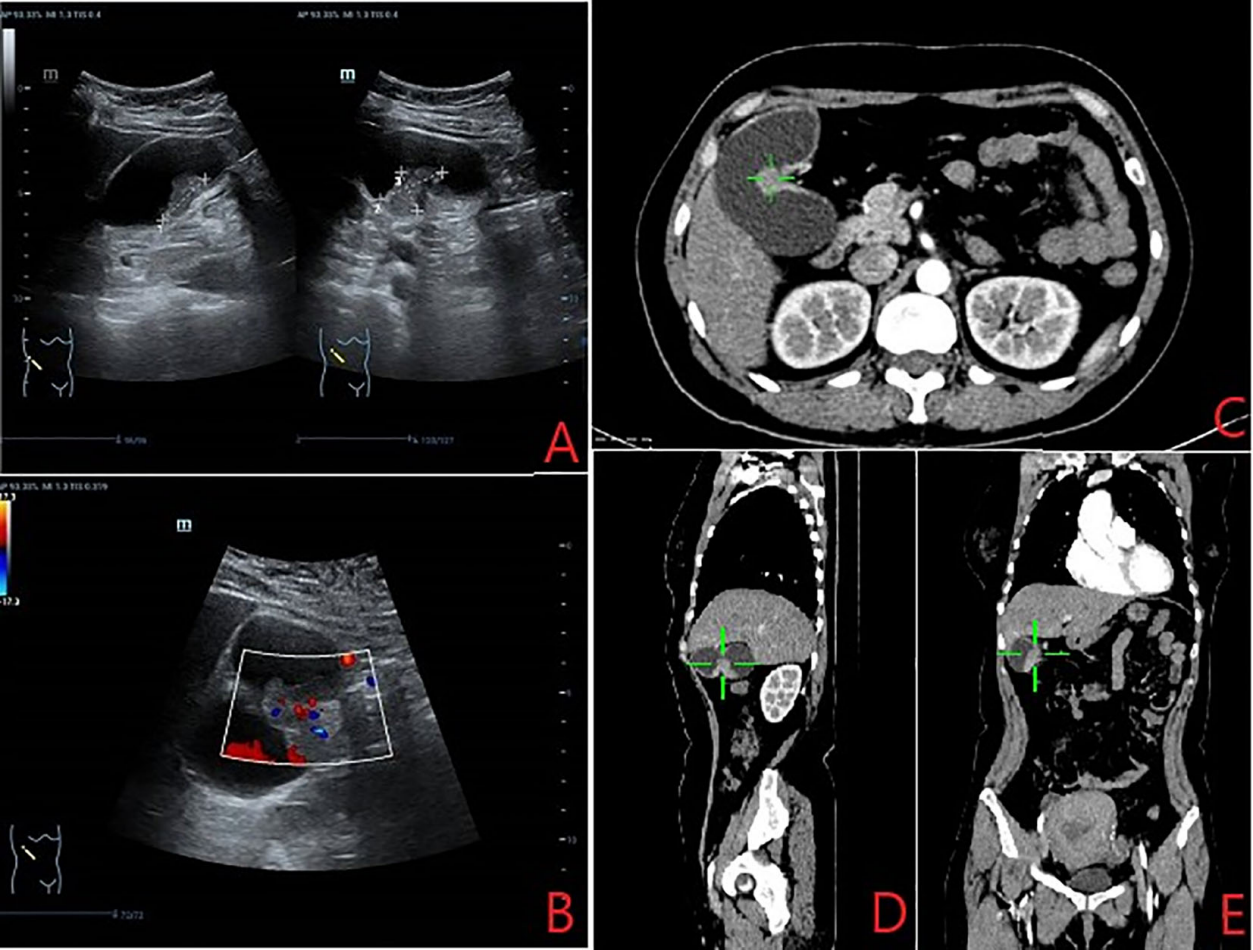
Preoperative imaging of gallbladder mixed adenoneuroendocrine carcinoma (GB-MANEC). **(A, B)** Abdominal ultrasonography. **(A)** Grayscale ultrasound revealed a heterogeneously isoechoic mass (calipers) occupying the gallbladder lumen, measuring approximately 3.0 cm × 2.5 cm × 2.0 cm, with an irregular contour. **(B)** Color Doppler imaging demonstrated prominent intralesional blood flow within the mass, indicative of hypervascularity. **(C–E)** Contrast-enhanced computed tomography (CT) viewed in three orthogonal planes. **(C)** The axial plane showed the tumor with irregular margins and heterogeneous enhancement (label “+”). **(D)** The sagittal plane further characterized the tumor’s morphology and its posterior extension (label “+”). **(E)** The coronal plane clearly delineated the extent of the mass (label “+”) and its relationship with the adjacent liver parenchyma.

Ultrasound first identified a polypoid mass and diffuse wall thickening within the gallbladder (**Figures 1A, B**). Subsequently, abdominal contrast-enhanced computed tomography (CT) scan revealed a gallbladder mass (approximately 3.0 cm × 2.5 cm × 2.0 cm) with irregular borders and heterogeneous enhancement. Notably, the mass displayed intracapsular growth and the absence of regional lymphadenopathy. These imaging findings were highly suspicious for an advanced gallbladder malignancy (**Figures 1C–E**).

#### 2.2.3 Surgical intervention

**Figure 2:**
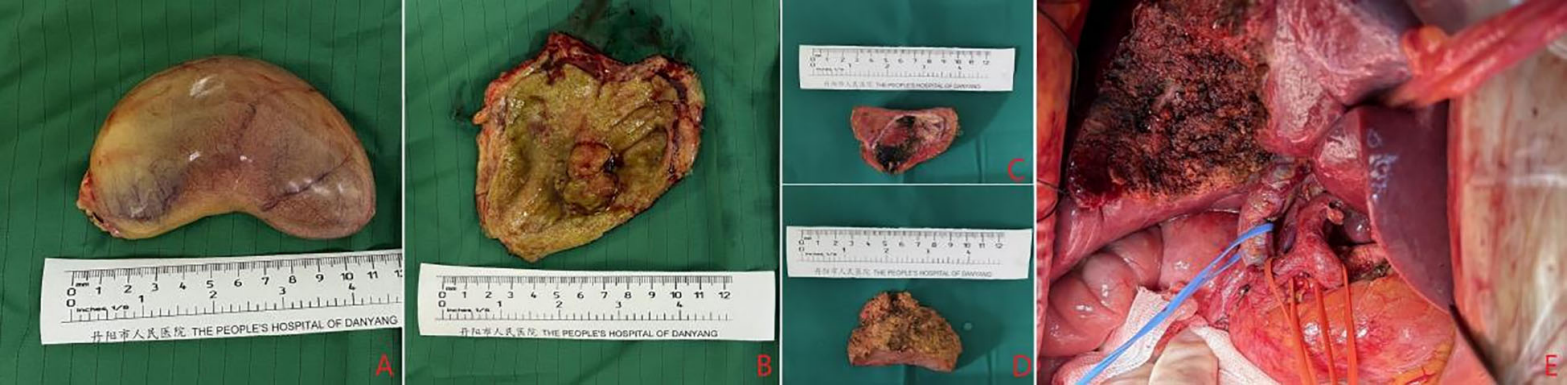
Gross pathological specimens following radical surgery for gallbladder mixed adenoneuroendocrine carcinoma (GB-MANEC). **(A, B)** Views of the resected gallbladder. **(A)** The external surface of the gallbladder appeared smooth. **(B)** The gallbladder was opened longitudinally, revealing a firm, whitish-tan tumor mass with ulcerated surfaces and infiltrative borders, located within the gallbladder lumen on the non-hepatic surface. **(C, D)** Views of the resected liver segments. **(C)** The hepatic resection margin at the gallbladder fossa was smooth and well-defined, and **(D)** no evidence of carcinoma infiltration was observed at the margin or within the adjacent hepatic parenchyma. **(E)** The complete radical cholecystectomy specimen encompassed the gallbladder, part of the liver segments, and the dissected regional lymph nodes (Stations 8, 12, and 13a). This panoramic view illustrated the en-bloc surgical principle for achieving oncological clearance.

Based on the preoperative clinical and radiological assessments, the patient initially underwent a cholecystectomy. Intraoperative inspection revealed a firm tumor measuring up to 3.0 cm in the greatest dimension and located within the gallbladder lumen on the non-hepatic surface, while the gallbladder serosa surface appeared smooth and free of tumor involvement. Intraoperative frozen section of the excised gallbladder revealed gallbladder adenocarcinoma (GB-AC) with a component of NEC. Consequently, a radical surgery consisting of cholecystectomy with an anatomical wedge resection of the liver parenchyma adjacent to the gallbladder fossa and a regional lymphadenectomy (Stations 8, 12, and 13a; **Figure 2E**) was performed. The operation was completed successfully without any immediate complications.

### 2.3 Pathological findings

#### 2.3.1 Gross examination

The resected specimen consisted of the gallbladder, a wedge of liver tissue, and regional lymph nodes. The gallbladder was opened longitudinally, revealing a firm, whitish-tan tumor mass with ulcerated surfaces and infiltrative borders, located within the gallbladder lumen on the non-hepatic surface and measuring approximately 3.0 cm × 2.5 cm × 2.0 cm. The gallbladder serosa surface appeared smooth, glistening, and free of tumor involvement (**Figures 2A, B**). The hepatic resection margin at the gallbladder fossa was smooth and well-defined, and no evidence of carcinoma infiltration was observed at the margin or within the adjacent hepatic parenchyma (**Figures 2C, D**).

#### 2.3.2 Histopathological analysis

**Figure 3:**
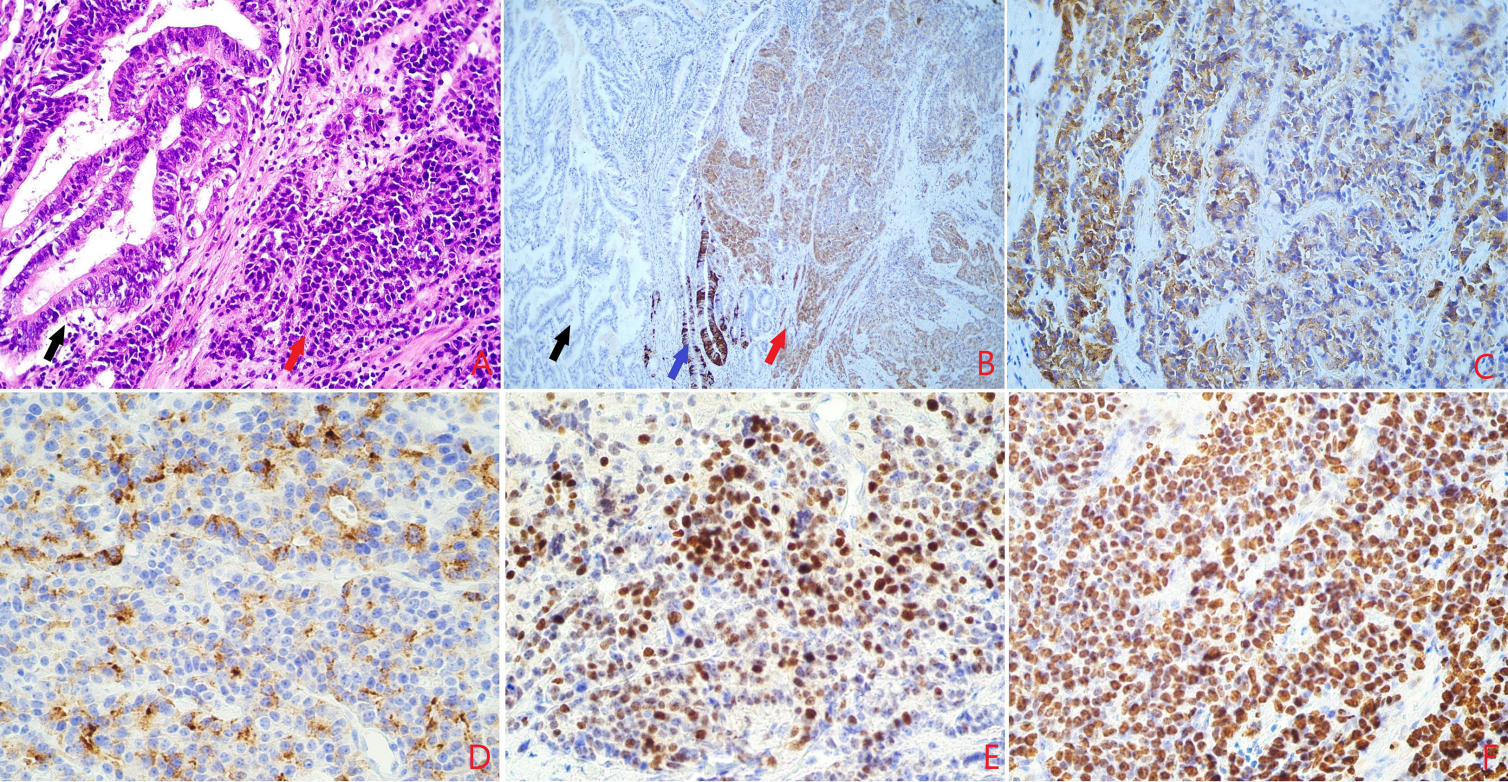
Histopathological and immunohistochemical features of gallbladder mixed adenoneuroendocrine carcinoma (GB-MANEC). **(A)** Hematoxylin and eosin (H&E) staining (×400) showed the biphasic nature of the tumor, including an adenocarcinoma component (black arrow) and a neuroendocrine tumor (NET) component (red arrow). **(B, C)** Immunohistochemical (IHC) staining for synaptophysin (Syn). The neuroendocrine carcinoma (NEC) component showed strong positive staining (red arrow), while the adenocarcinoma component was negative (black arrow). Notably, focal positive staining was also observed in some glandular structures at the transitional zone (blue arrow in panel **(B)**) (B, ×200; C, ×400). **(D)** IHC staining for chromogranin A (CgA; ×400) demonstrated positive expression in the NEC component. **(E)** IHC staining for insulinoma-associated protein 1 (INSM1; ×400) exhibited strong nuclear positivity in the NEC component. **(F)** IHC staining for Ki-67 (×400) showed a high proliferation index (>90%) in the neuroendocrine component, consistent with a grade 3 (G3) NEC.

Hematoxylin and eosin (H&E) staining revealed a biphasic tumor morphology with invasion of the serosal layer. One component, accounting for approximately 60% of the tumor, exhibited features of conventional GB-AC, characterized by glandular structures with cellular atypia. Adjacent to the adenocarcinoma, a second component, comprising approximately 40% of the tumor, was observed, consisting of cells arranged in solid nests, trabeculae, or rosette-like patterns with “salt-and-pepper” chromatin and a high mitotic rate (**Figure 3A**). The cystic duct and liver margins were both negative for carcinoma, and no vascular or perineural invasion was identified. A total of six regional lymph nodes were retrieved, none of which showed evidence of metastasis (0/6 positive). According to the eighth edition of the American Joint Committee on Cancer (AJCC) classification, the pathological TNM classification was pT3N0M0, resulting in a final pathological stage of Stage IIB. These findings indicate locally advanced disease with complete local resection (R0), but without regional lymphatic or distant metastasis.

#### 2.3.3 Immunohistochemistry

To confirm neuroendocrine differentiation, immunohistochemical staining was performed. Immunohistochemistry revealed that the neuroendocrine component exhibited strong and diffuse cytoplasmic positivity for synaptophysin (Syn), whereas the pure adenocarcinoma component was negative for Syn; notably, partial Syn expression was also observed in some adenocarcinoma cells within the transitional zone (**Figures 3B, C**). Furthermore, immunohistochemistry showed that chromogranin A (CgA) exhibited distinct granular cytoplasmic positivity (dot-like pattern) in the NEC cells (**Figure 3D**), while insulinoma-associated protein 1 (INSM1) demonstrated strong nuclear immunoreactivity (**Figure 3E**). Additionally, over 90% of the tumor cells were positive for Ki-67, indicating a very high proliferative rate in the neuroendocrine component, which is consistent with a high-grade NEC (G3) (**Figure 3F**).

### 2.4 Postoperative course and follow-up

The patient recovered well and was discharged in a stable condition. Postoperative high-throughput sequencing of the tumor identified a pathogenic amplification of the gene ERBB2 (HER2); gene mutations of TP53, NOTCH1, and KIT; and a gene fusion of SDHC (**Table 2**).

**Table 2:** The main results of high-throughput sequencing based on the MGI (DNBSEQ-T7) sequencing platform.

Detection items	Detection results		
Gene variants	Gene copy number variation	ERBB2	ERBB2 gene—4.5 copies—amplified
	Somatic mutations (single-nucleotide variants and small insertions/deletions)	TP53	p.D281N (frequency 56.78%)
		NOTCH1	p.L1756P (frequency 3.77%)
		KIT	p.F887V (frequency 1.49%)
	Gene fusion	SDHC	SDHC (exon 1–2)–ZNF519 (intergenic) fusion (frequency 0.96%)

The ERBB2 gene had a copy number value of approximately 4, which was significantly higher than the normal copy number of 2. The mutation frequencies of TP53 p.D281N, NOTCH1 p.L1756P, and KIT p.F887V were 56.78%, 3.77%, and 1.49%, respectively. The SDHC gene showed a clip at chr1:161293455, where the left side matched the SDHC gene sequence and the right side aligned with chr18:14068565. The frequency of SDHC (Exon 1–2)–ZNF519 (intergenic) fusion was 0.96%. Based on these findings, the patient received three cycles of adjuvant chemotherapy with a combination of etoposide, cisplatin, and gemcitabine, alongside the HER2-targeted monoclonal antibody trastuzumab. However, the treatment was complicated by progressive thrombocytopenia (31 × 10^9^/L, normal level, 125–350 × 10^9^/L) and progressive leukopenia (0.87 × 10^9^/L, normal level, 3.5–9.5 × 10^9^/L), accompanied by mild elevations in alkaline phosphatase (135.3 U/L, normal level, 50–135 U/L) and gamma-glutamyl transferase (55.1 U/L, normal level, 7–45 U/L) (**Table 1**). Consequently, adjuvant chemotherapy was discontinued, while the patient received two additional cycles of trastuzumab monotherapy. During the 7-month postoperative follow-up period, subsequent evaluations, including imaging and laboratory studies, demonstrated no signs of recurrence or metastasis.

## 3 Discussion

This case presented here exemplifies a rare and challenging malignancy. Based on the histopathological features, specifically the presence of both adenocarcinoma and NEC components, each exceeding 30%, this tumor was classified as a GB-MANEC according to the World Health Organization criteria (6, 7). This discussion can open an interesting perspective regarding early detection or risk stratification in rare aggressive gallbladder malignancies.

### 3.1 Characteristics, origin, and gene alteration

GB-MANECs, characterized by a dual differentiation of adenocarcinoma and neuroendocrine components, constitute a smaller subset of GB-NENs. The clinical presentation is notoriously non-specific, with symptoms such as abdominal pain, nausea, vomiting, and jaundice (10, 11). Less than 1% of patients with GB-NECs have carcinoid syndrome (15). This non-specificity frequently leads to a failure in preoperative diagnosis ^10^. The prognosis of MANEC frequently hinges on the most aggressive component within the tumor. Furthermore, the long-term prognosis of GB-NECs correlates with divergent tumor stage and degree of differentiation (11, 16, 17).

The origin of GB-MANEC remains unclear, particularly the origin of the neuroendocrine component. The main hypotheses about the origin of NEC in GB-MANEC are summarized as metaplasia–carcinogenesis, stem/progenitor cell derivation, and adenocarcinoma transdifferentiation (6, 18).

Due to the low incidence of gallbladder MANEC, research on the molecular basis of its pathogenesis remains relatively limited. Analysis based on somatic mutation/copy number alteration patterns revealed that the altered signaling pathways and associated genes in GBCs include the TP53 pathway (involving genes TP53 and MDM2), the cell cycle pathway (involving genes CDKN2A, CDKN2B, and RB1), the Receptor tyrosine kinase-RAS (RTK-RAS) pathway (involving genes KRAS, ERBB2, and ERBB3), the epigenetic regulation pathway (involving genes ARID1A and ARID2), the TGF-β pathway (involving the gene SMAD4), and the NOTCH pathway (involving the gene NOTCH1). Certain molecular alterations correlated with tumor histopathological characteristics (19). Variants in RB1 were enriched in tumors with small cell NEC (q < 0.001), and alterations in the cell cycle pathway were more common in non-adenocarcinoma tumors (e.g., adenocarcinoma 42% vs. small cell NEC 83%, q = 0.01) (20).

Based on the molecular differences of NETs and NECs, the mutations of MEN1, DAXX, and ATRX are entity-defining for well-differentiated NETs, whereas NECs usually have TP53 or RB1 mutations. MANECs show genomic alterations similar to those of adenocarcinomas or NECs (7).

In view of the genetic complexity of GB-MANEC, it is crucial to integrate multi-omics data for an in-depth exploration of the pathological mechanisms and therapeutic targets.

### 3.2 Diagnostic approach: from imaging to pathology

#### 3.2.1 Imaging limitations

Preoperative imaging with ultrasound and contrast-enhanced CT is crucial for identifying a gallbladder mass, assessing local invasion (e.g., into the liver), and detecting lymphadenopathy. However, these modalities cannot reliably distinguish MANEC from pure adenocarcinoma or other gallbladder malignancies based on imaging characteristics alone (3).

#### 3.2.2 Pathology as the “gold standard”

The definitive diagnosis of MANEC hinges on postoperative histopathological examination and immunohistochemistry (IHC). IHC is indispensable for confirming the neuroendocrine component, with markers such as Syn and CgA being present in up to 92.3% of patients, given their high sensitivity for diagnosing MANECs (11, 21). Additionally, INSM1, a transcriptional regulator with a characteristic zinc-finger DNA-binding domain, has been validated as a specific cytoplasmic marker of neuroendocrine differentiation (22). The Ki-67 proliferation index is a critical marker for grading the neuroendocrine component (G1–G3), which significantly influences prognosis and treatment decisions (7). According to the WHO grading system for NETs, the three grades of tumor differentiation are as follows: G1 (mitotic count <2 per 10 high power field (HPF) and/or Ki-67 index ≤2%), G2 (mitotic count 2–20 per 10 HPF and/or Ki-67 index 3%–20%), and G3 (mitotic count >20 per 10 HPF and/or Ki-67 index >20%) (7).

### 3.3 Management and evolving strategies

#### 3.3.1 Surgical cornerstone

Radical surgical resection (R0 resection) with adequate lymphadenectomy represents the cornerstone of curative-intent treatment for biliary NENs (23, 24). Overall, 74% of patients undergoing cholecystectomy for gallbladder NETs experience recurrence or metastasis. In contrast, cholecystectomy combined with hepatic segmentectomy and lymphadenectomy increases the 5-year survival rate from 21.3% to 60.4% (25).

For carcinoma *in situ* or tumors limited to the mucosa or submucosa, cholecystectomy alone is sufficient. For advanced cases without distant metastasis, cholecystectomy combined with en-bloc hepatic segmentectomy and a standard D2 lymphadenectomy (including Stations 8, 12, and 13a) is recommended (10, 26). Lymphadenectomy should include lymph nodes in the porta hepatis, gastrohepatic ligament, and retroduodenal regions (10, 27).

Several factors have been associated with worse recurrence-free survival (RFS) in gallbladder NECs: surgical resection type, margin status (R1 vs. R0, p = 0.001), advanced pathological AJCC stage (p < 0.0001), microscopic perineural invasion (p = 0.001), lymphovascular invasion (p < 0.0001), and positive lymph nodes (p = 0.001) (28). Complete R0 resection is an independent prognostic factor for overall survival and is critical for achieving a better prognosis in biliary NENs (23).

The prognostic significance of achieving R0 resection cannot be overstated. In a retrospective cohort of 28 biliary NEN patients, R0 resection margin was identified as an independent prognostic factor for overall survival (OS) in multivariate analysis (p = 0.027) (24). More recently, a nationwide multicenter study in China encompassing 36 surgically treated biliary NEN patients reported that a non-R0 margin was among the strongest independent risk factors for reduced OS, with a hazard ratio of 14.04 (95% CI: 2.67–73.79, p < 0.05) (29). This magnitude of effect underscores the critical importance of complete tumor extirpation.

The survival benefit of R0 resection is further corroborated by a large-scale analysis of the Surveillance, Epidemiology, and End Results (SEER) database, which demonstrated that the radical surgery approach was associated with improved OS in gallbladder NENs (p = 0.006) (24). A contemporary multicenter study reinforced these findings, providing level I evidence that R0 resection confers a substantial survival advantage in this patient population. Collectively, these data indicate that every effort should be made to achieve margin-negative resection, including extended resection, when necessary (29).

Regarding the comparative outcomes of R0 resection in GB-NEC versus gallbladder adenocarcinoma, available evidence suggests that despite more aggressive features of GB-NEC, the overall survival (p = 0.222) and disease-free survival (p = 0.269) were similar to those of gallbladder adenocarcinoma after curative surgery with negative resection margins, indicating comparable prognostic outcomes following R0 resection (30).

Equally critical is the performance of complete regional lymph node dissection. Lymph node metastasis (LNM) has consistently emerged as an adverse prognostic factor across multiple studies. In the SEER database analysis, LNM was identified as an independent prognostic factor for GB-NENs (p = 0.018), ampullary NENs (p = 0.006), and extrahepatic bile duct NENs (p = 0.036) (24). The National Cancer Database (NCDB) analysis of extrahepatic biliary NENs (n = 223) confirmed that lymph node metastases were associated with worse OS in the multivariable regression analysis [hazard ratio (HR) 1.19, 95% CI: 1.02–1.38] (31). Notably, while lymph node resection itself may not directly improve survival (p = 0.272 for gallbladder NENs), it is indispensable for accurate staging and risk stratification, as LNM status directly informs adjuvant therapy decisions and prognostic assessment (24).

In terms of lymphadenectomy quality, the AJCC and international expert consensus recommend the retrieval of at least six lymph nodes to ensure accurate staging and prognostic assessment (32). The majority of GB-NECs are detected at an advanced stage, with no specification regarding the status of resection margins in those undergoing surgery (33). Therefore, radical resection should be reserved for rigorously selected patients following multidisciplinary team (MDT) discussions. When deemed appropriate, the procedure must pursue R0 resection combined with regional lymph node dissection, adhering to contemporary clinical guidelines (30).

#### 3.3.2 The challenge of adjuvant therapy

Due to its extreme rarity and histological heterogeneity, there are no established adjuvant chemotherapy protocols for GB-MANEC, which makes it difficult to determine the effectiveness of adjuvant chemotherapy (11). However, adjuvant chemotherapy has been a treatment option for patients due to the high recurrence rate after surgery, with median survival rates ranging from 6 to 12 months (34). The primary treatment approach is generally formulated based on the characteristics of the NEC component at the time of MANEC diagnosis (11). There is evidence that the neuroendocrine component of the tumor may present a higher proliferative rate than the adenocarcinoma component (35). Compared with adenocarcinoma, the NEC component is considered more frequently associated with lymphovascular invasion, perineural invasion, and lymph node metastasis, exhibiting high aggressiveness and poor prognosis;while pure neuroendocrine components account for a significant proportion of lymph node metastases in MANEC patients; and deceased patients predominantly exhibited neuroendocrine components in either the primary tumor or metastatic lymph node (8, 9).

However, the grade of the adenocarcinoma component (HR 3.876, 95% CI: 1.451–10.357, p = 0.007) was an independent predictor of overall survival in the multivariate analysis. For intermediate-grade MANEC, treatment plans are primarily based on the adenocarcinoma component. Patients with well/moderately differentiated adenocarcinoma had a better prognosis, while for high-grade MANEC, treatment plans are determined according to the NEC component. Currently, platinum-based chemotherapy regimens (such as irinotecan plus cisplatin or etoposide plus cisplatin) are commonly used for the treatment of ampullary MANECs. Therefore, it is recommended that the optimal treatment for MANECs should take both the neuroendocrine and adenocarcinoma components into consideration (13, 14).

Treatment strategies are often extrapolated from protocols for adenocarcinoma or high-grade NEC. The option may depend on the dominant or higher-grade component (19). For the NEC component (often G3), regimens such as etoposide plus platinum are commonly considered (26, 36). For the adenocarcinoma component, or in mixed tumors without a clear dominant type, chemotherapy based on gemcitabine and platinum or other gallbladder adenocarcinoma regimens can be employed (13, 37).

### 3.4 Future directions and follow-up

Emerging research is exploring the potential of peptide receptor radionuclide therapy for NETs with high somatostatin receptor expression (38). Furthermore, advanced imaging analysis techniques, such as multimodal deep learning models that combine CT, somatostatin receptor PET, and laboratory biomarkers, are being investigated for their improved predictive value in patient survival, which can someday guide more personalized treatment plans (39–42).

A GB-MANEC patient received 10 cycles of 5-fluorouracil chemotherapy and radiotherapy after surgery, and the patient developed persistent low back pain after 6 months. CT confirmed retroperitoneal metastasis with multiple enlarged lymph nodes, prompting the discontinuation of radiotherapy and the initiation of somatostatin analogs (21).

Endocrine therapy for NENs is currently under intensive investigation due to the endocrine-dependent biological behavior of these tumor cells. Somatostatin analogs, including octreotide, lanreotide, and long-acting agent Sandostatin LAR, have a suppressive effect on the proliferation of tumor cells ^6^. Somatostatin analogs are capable of inhibiting tumor growth and stabilizing disease irrespective of the hormonal activity of the tumor; therefore, their applicability is expected to be extended to the treatment of hormonally inactive NETs as well (43).

A follow-up period of up to 10 years is recommended, particularly for patients with NEC components. Vigilance is required for potential NEC metastases even in cases with no recurrence for many years postoperatively. Similar to the case we have presented, fluctuations in tumor marker levels should prompt timely imaging investigations to detect early signs of recurrence or metastasis (44).

### 3.5 Prognosis and prognostic factors

The prognosis of GB-MANEC is generally unfavorable, primarily due to its unpredictable biological behavior. Literature has shown that the tumor-free survival and overall survival of biliary NETs are 5.8 (range 0.4–53.6) months and 13.7 (range 0.7–102.1) months, respectively, and the prognoses of NEC and MANEC are even worse (45). The key factors associated with a dismal outcome of GB-MANEC include a high-grade neuroendocrine component (the higher the Ki-67 index, the easier the tumor recurs and the poorer the prognosis, as seen in NEC G3) (46), the presence of lymph node metastasis (47), and higher TP53 mutations and the presence of RB1 mutations shown in GB-MANEC, which may be associated with shorter survival (45, 48), while the RB gene is known to inhibit cell proliferation, promote cell differentiation, and regulate the cell cycle (47).

This was demonstrated by our patient, who had no obvious abdominal symptoms except for unexplained anemia and was ultimately diagnosed with gallbladder malignancy. Anemia has emerged as an underappreciated but potentially valuable clinical marker in gallbladder malignancies (49). A study of 4,629 patients undergoing cholecystectomy found that preoperative anemia was significantly more common in patients with incidental gallbladder cancer (iGBC) compared to those with benign gallbladder disease; the hemoglobin levels differed significantly between the two groups (iGBC vs. benign, 12.5 ± 1.7 vs. 13.2 ± 1.9 g/dL; p = 0.007) (50). Furthermore, using a forward elimination model, Liava C. et al. found that preoperative anemia (OR 3.12; 95% CI: 1.60–6.11; p < 0.001) was one of six independent risk factors for identifying incidental GBC, with the other factors including maximum gallstone diameter ≥ 1.70 cm, age ≥ 68 years, female sex, and gallstone duration ≥ 5 years. Based on these factors, they constructed a complete GBC risk score formula, as follows: GBC risk score = (sex) × ln(4.155) + (age ≥ 68 years) × ln(4.549) + (gallstone diameter ≥ 1.70 cm) × ln(7.167) + (ascending cholangitis) × ln(5.347) + (gallstone duration ≥ 5 years) × ln(2.437) + (anemia) × ln(3.124). Combining anemia with other clinical parameters, such as advanced age (>65 years), can improve the pretest probability of identifying incidental malignancies (49, 50). The prognostic implications of anemia are less well-defined, but emerging evidence indicates that preoperative anemia may correlate with advanced disease stage and poorer postoperative outcomes. Anemia in cancer patients often reflects chronic inflammation, nutritional deficiency, or bone marrow involvement, all of which may portend a more aggressive clinical course (51).

In the context of rare and aggressive tumors such as GB-MANEC, where preoperative diagnosis is exceptionally challenging due to non-specific symptoms and imaging findings, the presence of otherwise unexplained anemia can serve as a low-cost, readily available adjunctive marker to raise clinical suspicion and potentially facilitate earlier detection.

## 4 Conclusion

GB-MANEC is a rare and aggressive malignancy, and the diagnosis relies heavily on comprehensive postoperative pathology and immunohistochemistry. While radical surgery offers the best chance for a cure, the overall prognosis remains unfavorable. The management of this disease requires a multidisciplinary approach, and the optimal adjuvant therapy regimen is yet to be defined. This case underscores the critical need for further research and the collection of more clinical data to establish standardized treatment protocols for this unusual tumor. Reporting individual cases and aggregating experiences in international registries are essential steps to improve outcomes for these patients.

## Data Availability

The original contributions presented in the study are included in the article/supplementary material. Further inquiries can be directed to the corresponding authors.

## References

[1] Roa JC García P Kapoor VK Maithel SK Javle M Koshiol J . Gallbladder cancer. Nat Rev Dis Primers. (2022) 8:69. doi: 10.1038/s41572-022-00398-y 36302789 PMC12314663

[2] Wang Z Wang L Hua Y Zhuang X Bai Y Wang H . Development and validation of a prognostic nomogram for gallbladder papillary adenocarcinoma. Front Oncol. (2023) 13:1157057. doi: 10.3389/fonc.2023.1157057 37260969 PMC10228726

[3] Ishikawa T Nakano K Osaka M Aratani K Yayoi K Akioka K . Mixed neuroendocrine-non-neuroendocrine neoplasms of the gallbladder: a case report. Surg Case Rep. (2021) 7:70. doi: 10.1186/s40792-021-01152-4 33730263 PMC7969674

[4] Wu X Li B Hong T Liu W He X Zheng C . Neuroendocrine neoplasm of the gallbladder: clinical features, surgical efficacy, and prognosis. Cancer Med. (2023) 12:11344–50. doi: 10.1002/cam4.5846 36952352 PMC10242308

[5] Díaz-López S Jiménez-Castro J Robles-Barraza CE Ayala-de Miguel C Chaves-Conde M . Mixed neuroendocrine non-neuroendocrine neoplasms in gastroenteropancreatic tract. World J Gastrointest Oncol. (2024) 16:1166–79. doi: 10.4251/wjgo.v16.i4.1166 38660639 PMC11037054

[6] Chu H Shi Y Liu J Huang D Zhang J Dou C . Update in clinical management for gallbladder neuroendocrine carcinoma. Med Baltimore. (2021) 100:e25449. doi: 10.1097/MD.0000000000025449 33832150 PMC8036038

[7] Nagtegaal ID Odze RD Klimstra D Paradis V Rugge M Schirmacher P . The 2019 WHO classification of tumours of the digestive system. Histopathology. (2020) 76:182–8. doi: 10.1111/his.13975 31433515 PMC7003895

[8] Chen H Shu M Chen S Xue L Lin Y . Clinicopathological features and lymph node metastatic patterns of gastric mixed adenoneuroendocrine carcinoma. Histol Histopathol. (2019) 34:373–9. doi: 10.14670/HH-18-045 30238962

[9] Zhang P Li Z Li J Li J Zhang X Lu Z . Clinicopathological features and lymph node and distant metastasis patterns in patients with gastroenteropancreatic mixed neuroendocrine-non-neuroendocrine neoplasm. Cancer Med. (2021) 10:5416–26. doi: 10.1002/cam4.4031 34109756 PMC8290235

[10] Zhang D Li P Szankasi P Liao X . Mixed adenoneuroendocrine carcinoma of the gallbladder, amphicrine type: case report and review of literature. Pathol Res Pract. (2020) 216:152997. doi: 10.1016/j.prp.2020.152997 32534704

[11] Machairas N Paspala A Frountzas M Tsilimigras DI Moris D Ntomi V . Mixed adenoneuroendocrine carcinoma (MANEC) of the gallbladder: a systematic review of outcomes following surgical management. In Vivo. (2019) 33:1721–6. doi: 10.21873/invivo.11662 31662496 PMC6899129

[12] Kalra N Gupta P Singhal M Gupta R Gupta V Srinivasan R . Cross-sectional imaging of gallbladder carcinoma: an update. J Clin Exp Hepatol. (2019) 9:334–44. doi: 10.1016/j.jceh.2018.04.005 31360026 PMC6637089

[13] Sung MK Lee W Hong S Park Y Kwak BJ Song KB . Mixed adenoneuroendocrine carcinoma of the ampulla of Vater: three case reports and a literature review. Ann Hepatobiliary Pancreat Surg. (2023) 27:107–13. doi: 10.14701/ahbps.22-054 36536502 PMC9947368

[14] Yang S Lu J Cai Y Li B Xiong X . Mixed adenoneuroendocrine carcinomas of stomach and ampulla of Vater after curative-intent resection: a single center case series. BMC Gastroenterol. (2021) 21:329. doi: 10.1186/s12876-021-01909-z 34433421 PMC8390255

[15] Zhang J Kulkarni HR Singh A Niepsch K Müller D Baum RP . Peptide receptor radionuclide therapy in grade 3 neuroendocrine neoplasms: safety and survival analysis in 69 patients. J Nucl Med. (2019) 60:377–85. doi: 10.2967/jnumed.118.215848 30115686

[16] de Mestier L Cros J Neuzillet C Hentic O Egal A Muller N . Digestive system mixed neuroendocrine-non-neuroendocrine neoplasms. Neuroendocrinology. (2017) 105:446–54. doi: 10.1159/000475527 28803232

[17] La Rosa S Marando A Sessa F Capella C . Mixed adenoneuroendocrine carcinomas (MANECs) of the gastrointestinal tract: an update. Cancers Bsl. (2012) 4:11–26. doi: 10.3390/cancers4010011 24213223 PMC3712682

[18] Kong FH Xu GD Liu CQ Ma H Zou YX Li XF . Mixed adenoneuroendocrine carcinoma of the gallbladder: a case report and literature review. Front Oncol. (2025) 15:1584744. doi: 10.3389/fonc.2025.1584744 40823077 PMC12350106

[19] Sanchez-Vega F Mina M Armenia J Chatila WK Luna A La KC . Oncogenic signaling pathways in the cancer genome atlas. Cell. (2018) 173:321–337.e10. doi: 10.1016/j.cell.2018.03.035 29625050 PMC6070353

[20] Giraldo NA Drill E Satravada BA El Dika I Brannon AR Dermawan J . Comprehensive molecular characterization of gallbladder carcinoma and potential targets for intervention. Clin Cancer Res. (2022) 28:5359–67. doi: 10.1158/1078-0432.CCR-22-1954 36228155 PMC9772093

[21] Costa AC Cavalcanti CLC Coelho HGB Leão LHA Soares DTC Santa-Cruz F . Rare mixed adenoneuroendocrine carcinoma of the gallbladder: case report and review of literature. Am J Case Rep. (2021) 22:e929511. doi: 10.12659/AJCR.929511 33945521 PMC8105744

[22] Mahalakshmi B Baskaran R Shanmugavadivu M Nguyen NT Velmurugan BK . Insulinoma-associated protein 1 (INSM1): a potential biomarker and therapeutic target for neuroendocrine tumors. Cell Oncol Dordr. (2020) 43:367–76. doi: 10.1007/s13402-020-00505-9 32219703 PMC12990676

[23] Zheng Z Chen C Li B Liu H Zhou L Zhang H . Biliary neuroendocrine neoplasms: clinical profiles, management, and analysis of prognostic factors. Front Oncol. (2019) 9:38. doi: 10.3389/fonc.2019.00038 30805307 PMC6370735

[24] Zheng BH Zhang C Wan WZ Sun WT Cheng X Ni XJ . The clinical and prognostic factors for biliary neuroendocrine neoplasm: a study based on the SEER database. BMC Surg. (2022) 22:253. doi: 10.1186/s12893-022-01689-7 35768809 PMC9245279

[25] Song W Chen W Zhang S Peng J He Y . Successful treatment of gallbladder mixed adenoneuroendocrine carcinoma with neo-adjuvant chemotherapy. Diagn Pathol. (2012) 7:163. doi: 10.1186/1746-1596-7-163 23186166 PMC3584922

[26] Shindoh J de Aretxabala X Aloia TA Roa JC Rocha FG Croome K . International consensus on gallbladder cancer: a report from the IHPBA-APHPBA consensus meeting on gallbladder cancer. HPB Oxford. (2022) 24:1996–2007. doi: 10.1016/j.hpb.2022.08.005 36443197

[27] Benson AB Abrams TA Ben-Josef E Bloomston PM Botha JF Clary BM . NCCN clinical practice guidelines in oncology: hepatobiliary cancers. J Natl Compr Canc Netw. (2009) 7:350–91. doi: 10.6004/jnccn.2009.0027 19406039 PMC4461147

[28] Hu YF Wang JK Ma WJ Hu HJ Gu HF Liu F . Does the size of the neuroendocrine-carcinoma component determine the prognosis of gallbladder cancer? Front Endocrinol Lausanne. (2024) 15:1217250. doi: 10.3389/fendo.2024.1217250 39104815 PMC11298461

[29] Wang W Yang CX Yu XZ Zhang SL Wang J Wang J . Clinicopathological characteristics and prognostic factors of patients with primary gallbladder neuroendocrine carcinomas. J Dig Dis. (2022) 23:107–14. doi: 10.1111/1751-2980.13088 35187836

[30] Tidjane A Bouzid C Berkane S Serradj NB Bendjebbar K Krelil B . Comparative survival analysis of gallbladder neuroendocrine carcinoma and adenocarcinoma following radical surgery: a secondary analysis of the multicenter Algerian GBC group study. Surg Oncol Insight. (2025) 2:100195. doi: 10.1016/j.soi.2025.100195 38826717

[31] Dominguez DA Eade AV Aversa JG Hagerty BL Blakely AM Davis JL . Extrahepatic biliary neuroendocrine tumors: a national cancer database analysis. Heliyon. (2024) 10:e34714. doi: 10.1016/j.heliyon.2024.e34714 39144996 PMC11320154

[32] Amin MB Greene FL Edge SB Compton CC Gershenwald JE Brookland RK . The eighth edition AJCC cancer staging manual: continuing to build a bridge from a population-based to a more "personalized" approach to cancer staging. CA Cancer J Clin. (2017) 67:93–9. doi: 10.3322/caac.21388 28094848

[33] Chen C Wang L Liu X Zhang G Zhao Y Geng Z . Gallbladder neuroendocrine carcinoma: report of 10 cases and comparison of clinicopathologic features with gallbladder adenocarcinoma. Int J Clin Exp Pathol. (2015) 8:8675–83. PMC4555718 26339390

[34] Millet C Farokhian A Mekheal N Singh B Baddoura W . Massive mixed adenoneuroendocrine carcinoma: a case report. Cureus. (2021) 13:e15928. doi: 10.7759/cureus.15928 34258127 PMC8255114

[35] Shimizu T Tajiri T Akimaru K Arima Y Yoshida H Yokomuro S . Combined neuroendocrine cell carcinoma and adenocarcinoma of the gallbladder: report of a case. J Nippon Med Sch. (2006) 73:101–5. doi: 10.1272/jnms.73.101 16641536

[36] Sorbye H Baudin E Borbath I Caplin M Chen J Cwikla JB . Unmet needs in high-grade gastroenteropancreatic neuroendocrine neoplasms (WHO G3). Neuroendocrinology. (2019) 108:54–62. doi: 10.1159/000493318 30153658

[37] Valle J Wasan H Palmer DH Cunningham D Anthoney A Maraveyas A . Cisplatin plus gemcitabine versus gemcitabine for biliary tract cancer. N Engl J Med. (2010) 362:1273–81. doi: 10.1056/NEJMoa0908721 20375404

[38] Strosberg J EI-Haddad G Wolin E Hendifar A Yao J Chasen B . Phase 3 trial of 177Lu-dotatate for midgut neuroendocrine tumors. N Engl J Med. (2017) 376:125–36. doi: 10.1056/NEJMoa1607427 28076709 PMC5895095

[39] Zhang J Jiang X Jiang H Gu Y Gao Y Guo Y . Development and validation of a deep learning model for survival prediction in patients with neuroendocrine neoplasms using multimodal data. Eur J Nucl Med Mol Imaging. (2024) 51:1164–75. doi: 10.1007/s00259-023-06546-0 38095672

[40] Yang Z Han Y Li F Zhang A Cheng M Gao J . Deep learning radiomics analysis based on computed tomography for survival prediction in gastric neuroendocrine neoplasm: a multicenter study. Quant Imaging Med Surg. (2023) 13:8190–203. doi: 10.21037/qims-23-577 38106311 PMC10721996

[41] Prisciandaro M Antista M Raimondi A Corti F Morano F Centonze G . Biomarker landscape in neuroendocrine tumors with high-grade features: current knowledge and future perspective. Front Oncol. (2022) 12:780716. doi: 10.3389/fonc.2022.780716 35186729 PMC8856722

[42] Campanale D Imperiale A Albano D Rizzo A Piccardo A Treglia G . Detection of cardiac neuroendocrine tumour metastases by somatostatin receptor PET/CT: a systematic review and meta-analysis. Front Med Lausanne. (2024) 11:1491181. doi: 10.3389/fmed.2024.1491181 39473497 PMC11518709

[43] Igaz P . Efficacy of somatostatin analogues in the treatment of neuroendocrine tumours based on the results of recent clinical trials. Orv Hetil. (2014) 155:1943–7. doi: 10.1556/OH.2014.30048 25417137

[44] Quanwei G Yiqiang Y Gangpu W . Case report: postoperative cervical lymph node metastasis of the neuroendocrine carcinoma component of rectal mixed adenoneuroendocrine carcinoma. Front Oncol. (2025) 15:1464426. doi: 10.3389/fonc.2025.1464426 40909957 PMC12404991

[45] Liu S Zhong Z Xiao M Song Y Zhu Y Hu B . Mixed adenoneuroendocrine carcinoma of the hepatic bile duct: a case report and review of the literature. BMC Gastroenterol. (2020) 20:399. doi: 10.1186/s12876-020-01550-2 33238879 PMC7691051

[46] Kim J Lee WJ Lee SH Lee KB Ryu JK Kim YT . Clinical features of 20 patients with curatively resected biliary neuroendocrine tumours. Dig Liver Dis. (2011) 43:965–70. doi: 10.1016/j.dld.2011.07.010 21856258

[47] Yang JJ Li ZP Luo CL Du Y Lu QY Li N . Mixed adenoneuroendocrine carcinoma of the liver: a rare case report. Mol Clin Oncol. (2020) 12:148–54. doi: 10.3892/mco.2019.1962 31929886 PMC6951250

[48] Song IH Ahn B Park YS Kim DH Hong SM . Presence of RB1 or absence of LRP1B mutation predicts poor overall survival in patients with gastric neuroendocrine carcinoma and mixed adenoneuroendocrine carcinoma. Cancer Res Treat. (2025) 57:492–506. doi: 10.4143/crt.2024.667 39327909 PMC12016830

[49] Liava C Starlinger P Kamath PS Hilscher MB . Risk factors for incidental gallbladder cancer found during cholecystectomy for cholelithiasis: a case–control study. Gastro Hep Adv. (2025) 4:100655. doi: 10.1016/j.gastha.2025.100655 40487276 PMC12137155

[50] Ahn Y Park CS Hwang S Jang HJ Choi KM Lee SG . Incidental gallbladder cancer after routine cholecystectomy: when should we suspect it preoperatively and what are predictors of patient survival? Ann Surg Treat Res. (2016) 90:131–8. doi: 10.4174/astr.2016.90.3.131 26942156 PMC4773457

[51] Miglietta F Pirozzi M Bottosso M Pisani C Franco P Guarneri V . Anaemia in cancer patients: advances and challenges in the era of precision oncology. Crit Rev Oncol Hematol. (2025) 204:104788. doi: 10.1016/j.critrevonc.2025.104788 40466818

